# Supplementary material for: Heparin-based hydrogel scaffolding alters the transcriptomic profile and increases the chemoresistance of MDA-MB-231 triple-negative breast cancer cells
Source: Biomater Sci. 2020 Feb 13;8(10):2786–96. doi: 10.1039/c9bm01481k (PMC7497406; doi:10.1039/c9bm01481k)
Supplement: Supplementary file 2 [file BM-008-C9BM01481K-s002.zip › Supplementary File 4/HP-B hydrogel + EGF (s)vControl/Pathways/my_analysis.Gsea.1545201170987/HALLMARK_ANGIOGENESIS.html]

Details for gene set HALLMARK\_ANGIOGENESIS[GSEA]

|  || Dataset | expr.class.cls#HYDEGF\_versus\_CONTROL.class.cls#HYDEGF\_versus\_CONTROL\_repos |
| Phenotype | class.cls#HYDEGF\_versus\_CONTROL\_repos |
| Upregulated in class | CONTROL |
| GeneSet | HALLMARK\_ANGIOGENESIS |
| Enrichment Score (ES) | -0.37292525 |
| Normalized Enrichment Score (NES) | -1.187927 |
| Nominal p-value | 0.22045454 |
| FDR q-value | 0.16673043 |
| FWER p-Value | 0.944 |
Table: GSEA Results Summary

  

Fig 1: Enrichment plot: HALLMARK\_ANGIOGENESIS      
 Profile of the Running ES Score & Positions of GeneSet Members on the Rank Ordered List

  

| PROBE | DESCRIPTION (from dataset) | GENE SYMBOL | GENE\_TITLE | RANK IN GENE LIST | RANK METRIC SCORE | RUNNING ES | CORE ENRICHMENT || 1 | PDGFA | na |  |  | 813 | 1.811 | 0.0327 | No |
| 2 | VAV2 | na |  |  | 1446 | 1.450 | 0.0598 | No |
| 3 | FGFR1 | na |  |  | 2109 | 1.236 | 0.0765 | No |
| 4 | COL5A2 | na |  |  | 2434 | 1.144 | 0.1071 | No |
| 5 | PTK2 | na |  |  | 2705 | 1.079 | 0.1377 | No |
| 6 | JAG2 | na |  |  | 3263 | 0.949 | 0.1481 | No |
| 7 | LRPAP1 | na |  |  | 5331 | 0.599 | 0.0651 | No |
| 8 | VCAN | na |  |  | 7533 | 0.301 | -0.0372 | No |
| 9 | VTN | na |  |  | 7622 | 0.286 | -0.0299 | No |
| 10 | PGLYRP1 | na |  |  | 7669 | 0.281 | -0.0207 | No |
| 11 | FSTL1 | na |  |  | 8207 | 0.222 | -0.0395 | No |
| 12 | LPL | na |  |  | 11671 | -0.184 | -0.2125 | No |
| 13 | THBD | na |  |  | 14480 | -0.553 | -0.3360 | No |
| 14 | TNFRSF21 | na |  |  | 14534 | -0.561 | -0.3155 | No |
| 15 | CCND2 | na |  |  | 14645 | -0.580 | -0.2972 | No |
| 16 | JAG1 | na |  |  | 16098 | -0.843 | -0.3380 | Yes |
| 17 | TIMP1 | na |  |  | 16662 | -0.990 | -0.3263 | Yes |
| 18 | NRP1 | na |  |  | 17279 | -1.174 | -0.3098 | Yes |
| 19 | SERPINA5 | na |  |  | 17287 | -1.177 | -0.2614 | Yes |
| 20 | APP | na |  |  | 18704 | -2.052 | -0.2501 | Yes |
| 21 | ITGAV | na |  |  | 18729 | -2.091 | -0.1647 | Yes |
| 22 | S100A4 | na |  |  | 18812 | -2.210 | -0.0774 | Yes |
| 23 | STC1 | na |  |  | 18901 | -2.351 | 0.0155 | Yes |
Table: GSEA details [plain text format]

  

Fig 2: HALLMARK\_ANGIOGENESIS      
 Blue-Pink O' Gram in the Space of the Analyzed GeneSet

  

Fig 3: HALLMARK\_ANGIOGENESIS: Random ES distribution      
 Gene set null distribution of ES for **HALLMARK\_ANGIOGENESIS**
